# Three new pentatricopeptide repeat proteins facilitate the splicing of mitochondrial transcripts and complex I biogenesis in Arabidopsis

**DOI:** 10.1093/jxb/ery275

**Published:** 2018-07-25

**Authors:** Chuande Wang, Fabien Aubé, Martine Quadrado, Céline Dargel-Graffin, Hakim Mireau

**Affiliations:** 1 Institut Jean-Pierre Bourgin, INRA, AgroParisTech, CNRS, Université Paris-Saclay, Versailles Cedex, France; 2 Paris-Sud University, Université Paris-Saclay, Orsay Cedex, France

**Keywords:** *Arabidopsis thaliana*, complex I biogenesis, mitochondria, NADH dehydrogenase, PPR, splicing

## Abstract

Group II introns are common features of most angiosperm mitochondrial genomes. Intron splicing is thus essential for the expression of mitochondrial genes and is facilitated by numerous nuclear-encoded proteins. However, the molecular mechanism and the protein cofactors involved in this complex process have not been fully elucidated. In this study, we characterized three new pentatricopeptide repeat (PPR) genes, called *MISF26*, *MISF68*, and *MISF74*, of Arabidopsis and showed they all function in group II intron splicing and plant development. The three PPR genes encode P-type PPR proteins that localize in the mitochondrion. Transcript analysis revealed that the splicing of a single intron is altered in *misf26* mutants, while several mitochondrial intron splicing defects were detected in *misf68* and *misf74* mutants. To our knowledge, MISF68 and MISF74 are the first two PPR proteins implicated in the splicing of more than one intron in plant mitochondria, suggesting that they may facilitate splicing differently from other previously identified PPR splicing factors. The splicing defects in the *misf* mutants induce a significant decrease in complex I assembly and activity, and an overexpression of mRNAs of the alternative respiratory pathway. These results therefore reveal that nuclear encoded proteins MISF26, MISF68, and MISF74 are involved in splicing of a cohort of mitochondrial group II introns and thereby required for complex I biogenesis.

## Teaser

Three nuclear encoded multi-functional pentatricopeptide repeat proteins localizing in the mitochondrion are involved in splicing of a cohort of mitochondrial group II introns and thereby required for complex I biogenesis.

## Introduction

Mitochondria are essential eukaryotic organelles that are responsible for energy production via aerobic respiration and are thought to have originated from the endosymbiotic association of an ancient α-proteobacterium (Gray *et al.*, 1999; Andersson *et al.*, 2003; Gray, 2012). Over the course of evolution, most genes that were present in the original endosymbiont have been transferred to the nucleus of the host cell, resulting in a significant reduction of the mitochondrial gene content (Martin and Herrmann, 1998). Nonetheless, a small proportion of the ancestral genes have been retained in modern mitochondria. In plants, mitochondrial genomes exhibit considerable variations in size, structure and organization (Kubo and Newton, 2008; Gualberto and Newton, 2017). Plant mitochondrial genomes are highly recombinogenic and complex RNA expression processes have likely evolved in response (Hammani and Giegé, 2014). Among these, the splicing of numerous introns, which interrupt protein-coding genes, is required to generate translatable mature mRNAs and is accomplished for the most part by nuclear-encoded protein *trans*-factors (Bonen, 2008; Colas des Francs-Small and Small, 2014; Hammani and Giegé, 2014). As observed in mutants affected in the genes encoding such *trans*-factors, dysfunction in the removal of plant mitochondrial introns often has severe consequences for plant fitness (Brown *et al.*, 2014; Colas des Francs-Small and Small, 2014; Hammani and Giegé, 2014).

Based on RNA structures and splicing mechanisms, mitochondrial introns can be classified into two families and in seed plants most of them are categorized as group II. A total of 23 group II introns have been identified in Arabidopsis mitochondria, requiring either *cis*-splicing or *trans*-splicing reactions for their removal (Bonen, 2008). Classical group II introns share a conserved structure consisting of six helical domains (DI–DVI) that radiate from a central hub (Bonen, 2008; Glanz and Kuck, 2009). The DV and DVI domains are especially important for the splicing reactions. DV is a highly conserved domain comprising the intron catalytic core, and DVI contains an internal bulged adenosine that acts as the attacking nucleophile to initiate the splicing reaction (Zimmerly and Semper, 2015). At least two splicing pathways for group II introns have been identified in seed plants (Li-Pook-Than and Bonen, 2006; Gualberto *et al.*, 2015; Massel *et al.*, 2016). The ‘branching’ pathway occurs via a two-step *trans*-esterification reaction. In the first step, the 2′-OH group of the branch point adenosine attacks the phosphate at the 5′-splice site. The 5′-exon is released and the intron, still attached to the 3′-exon, adopts a branched structure giving the intron a lariat form. In the second step, the 3′-OH of the 5′-exon attacks the 3′-splice site, resulting in ligated exons and the release of the intron as a lariat with a tiny tail (Brown *et al.*, 2014). An alternative splicing pathway, known as the ‘hydrolytic pathway’, generates a linear intron and was adopted by some group II introns whose certain conserved features have been lost during the course of evolution (Li-Pook-Than and Bonen, 2006; Gualberto *et al.*, 2015; Massel *et al.*, 2016).

While some group II introns can undergo autocatalytic (i.e. self-) splicing *in vitro*, all are thought to require one or more proteins *in vivo*, such as intron-encoded maturases. Plant organellar group II introns are highly degenerate and lack regions that are essential for self-splicing. MatR is the only intron-encoded maturase that has been maintained in seed plant mitochondria (Wahleithner *et al.*, 1990), and a recent study indicated that it is associated with splicing of multiple mitochondrial introns *in vivo* (Sultan *et al.*, 2016). Four *matR* homologs (*nMAT1*–*nMAT4*) reside in the nuclear genome of angiosperms and their participation in the splicing of mitochondrial group II introns has been established (Keren *et al.*, 2009, 2012; Cohen *et al.*, 2014; Zmudjak *et al.*, 2017). Other nuclear-encoded cofactors facilitating mitochondrial intron splicing include RUG3 (RCC1/UVR8/GEF-like 3), mTERF15 (Mitochondrial Transcription Termination Factor 15), WTF9 (What’s This Factor? 9), mCSF1 (Mitochondrial CAF-like Splicing Factor 1), ABO6 (ABA Overly Sensitive 6) and PMH2 (Putative Mitochondrial RNA Helicase 2) (Köhler *et al.*, 2010; Kuhn *et al.*, 2011; Francs-Small *et al.*, 2012; He *et al.*, 2012; Zmudjak *et al.*, 2013; Hsu *et al.*, 2014; Zmudjak *et al.*, 2017). Recently, the RAD52-like protein ODB1, which was originally identified as a mitochondrial DNA repair component, has also been shown to be required for the efficient splicing of two group II introns (Gualberto *et al.*, 2015). Aside from these splicing factors, the pentatricopeptide repeat (PPR) proteins are by far the most represented class of proteins playing roles in the splicing of mitochondrial introns (Barkan and Small, 2014). PPR proteins are composed of 2–30 tandem repeats of highly degenerate 35-amino-acid motifs that fold into a pair of antiparallel α-helices (Ke *et al.*, 2013; Yin *et al.*, 2013; Barkan and Small, 2014; Shen *et al.*, 2016). Based on the nature of their repeats, PPR proteins can be divided into two major subfamilies (Lurin *et al.*, 2004). The PLS-type PPR proteins contain triplets of P, L (Long), and S (Short) motifs and are almost exclusively implicated in RNA editing in plant organelles (Takenaka *et al.*, 2013*b*; Sun *et al.*, 2016). P-type PPR proteins contain only canonical 35-amino-acid repeats and take part in a wide range of post-transcriptional processes ranging from RNA maturation to translation in organelles (reviewed in Barkan and Small, 2014). Several PPR proteins implicated in RNA splicing have been characterized in Arabidopsis, such as OTP43 (Organelle Transcript Processing defect 43), ABO5 (ABA Overly Sensitive 5), ABO8 (ABA Overly Sensitive 8), BIR6 (Buthionine Sulfomixine-Insensitive Roots 6), OTP439 (Organelle Transcript Processing 439), and TANG2 or SLO3 (Slow Growth 3). Each of these proteins is highly specific and required for the splicing of a single mitochondrial intron in most cases (Colas des Francs-Small *et al.*, 2014; de Longevialle *et al.*, 2007; Koprivova *et al.*, 2010; Liu *et al.*, 2010; Yang *et al.*, 2014; Hsieh *et al.*, 2015). Taken together, these studies demonstrated the important role of PPR proteins in RNA splicing of plant mitochondria, but the role they play in this process is currently unknown.

In this study, we report the function of three newly characterized mitochondria-targeted PPR proteins, named Mitochondrial Intron Splicing Factor 26 (MISF26), Mitochondrial Intron Splicing Factor 68 (MISF68) and Mitochondrial Intron Splicing Factor 74 (MISF74), which are essential for the splicing of either one or several mitochondrial introns in Arabidopsis. The loss-of-function of these nuclear-encoded splicing factors affects complex I assembly, increases the expression of alternative oxidase genes, and results in retarded plant growth.

## Materials and methods

### Plant material

Arabidopsis Col-0 plants were obtained from the Arabidopsis Stock Centre of the Institut National de la Recherche Agronomique in Versailles (http://publiclines.versailles.inra.fr/). Arabidopsis T-DNA mutants N508252 (*misf26-1*), N816920 (*misf26-2*), N562109 (*misf68-1*), N510785 (*misf68-2*), and N554210 (*misf74-1*) were obtained from the European Arabidopsis Stock Centre (http://arabidopsis.info/), and N191C05 (*misf74-2*) was acquired from the GABI-Kat mutant collection (https://www.gabi-kat.de/). Plants homozygous for the insertions were identified by PCR genotyping. Specific primers used for genotyping are listed in Supplementary Table S1 at *JXB* online. Plants were grown on soil in a greenhouse under long-day conditions (16 h of light and 8 h of dark).

### Subcellular localization of MISF26, MISF68, and MISF74

DNA regions encoding the N-terminal targeting sequence of each MISF protein were PCR amplified, cloned into pDONR207 by Gateway^TM^ BP reaction (Invitrogen) and sequenced to check PCR accuracy. The obtained entry clones were subsequently transferred into pGWB5 (Nakagawa *et al.*, 2007) by the Gateway^TM^ LR reaction (Invitrogen) to create a translational fusion between the targeting sequences and the coding region of green fluorescent protein (GFP). The fusion constructs were transformed into *Agrobacterium tumefaciens* C58C51 and used for floral dip transformation of Arabidopsis Col-0 plants. Transgenic plants were selected on hygromycin, and GFP fluorescence was visualized in root cells by Leica TCS SP2 confocal microscopy. Prior to observation, roots were soaked in a solution of Mitotracker^TM^ Red (0.1 μM) to label mitochondria.

### RNA extraction and analysis

Total RNA was extracted from 8-week-old Arabidopsis flower buds using TRIzol reagent (Life Technologies) following the manufacturer’s instructions and treated with RTS DNase (Mobio) only when RNAs were prepared for reverse transcription (RT)-PCR or quantitative RT-PCR (qRT-PCR). RT-PCR analysis was performed to check the expression of *MISF* genes using primer pairs listed in Supplementary Table S1. The *BIO2* gene was used as control to check cDNA integrity. RNA gel blot analyses were performed as described in (Haïli *et al.*, 2016). Specific probes were amplified by PCR using primers indicated in Supplementary Table S1. To quantify the splicing efficiency of mitochondrial introns, quantitative RT-PCR was performed using primers designed across intron–exon regions (unspliced forms) and exon–exon junctions (spliced forms) as previously described (Koprivova *et al.*, 2010; Haïli *et al.*, 2013). Two biological and three technical repeats were performed and the nuclear 18S ribosomal RNA gene was used for data normalization.

### Blue Native gel and in-gel activity assays

Eight-week-old Arabidopsis flower buds were used to prepare crude mitochondrial extracts as previously described (Dahan *et al.*, 2014). One hundred micrograms of total proteins from purified organelles was loaded and separated on 4–16% (w/v) polyacrylamide NativePAGE^TM^ Bis/Tris gels (Invitrogen). Following electrophoresis, Blue Native (BN)-PAGE gels were transferred to polyvinylidene difluoride (PVDF) membranes or subjected to a complex I in-gel activity assay (Wang *et al.*, 2017*a*). Succinctly, complex I activity was revealed by incubating gels in the presence of 0.2% nitroblue tetrazolium, 0.2 mM NADH and 0.1 M Tris–HCl (pH 7.4). When sufficient coloration was obtained, gels were soaked in fixing solution containing 30% (v/v) methanol and 10% (v/v) acetic acid to stop the reactions. For immuno-detection, BN-PAGE gels were transferred overnight to PVDF membranes in transfer buffer (50 mM Bis/Tris and 50 mM Tricine) at 30 V and at 4 °C. Membranes were then hybridized with antibodies to carbonic anhydrase (Cohen *et al.*, 2014). Hybridization signals were revealed using enhanced chemiluminescence reagents (Western Lightning Plus ECL, Perkin Elmer).

## Results

### Three *ppr* mutants displaying slow-growth phenotypes

**Fig. 1.**
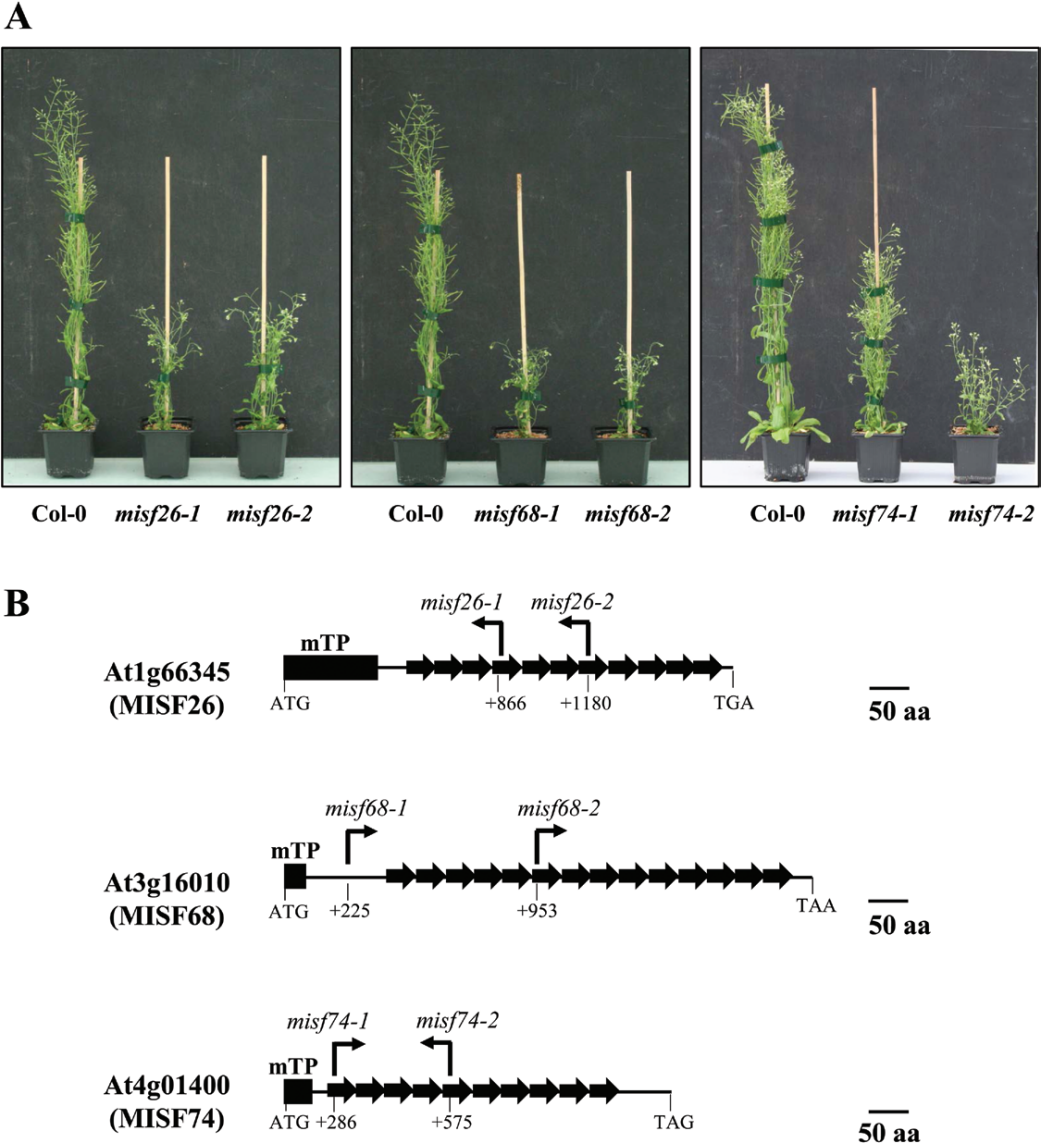
The three identified Arabidopsis *misf* mutants display strongly delayed developmental phenotypes. (A) Eight-week-old plants showing the global vegetative phenotype of the *misf* mutants compared with the wild-type (Col-0). (B) Schematic diagram of MISF26, MISF68, and MISF74 proteins depicting the 11, 14, and 10 predicted PPR-P repeats that they contain, respectively. The locations of corresponding T-DNA insertion sites are indicated. The putative mitochondrial targeting sequences (mTP) are shown as black boxes and were predicted using TargetP.

In an effort to better understand mitochondrial gene expression in plants, we assembled a large collection of Arabidopsis T-DNA insertion mutants disrupted in nuclear genes encoding putative mitochondria-targeted PPR proteins. Among them, we found three mutants showing global growth retardation phenotypes compared with wild-type plants, and that resemble previously identified Arabidopsis complex I mutants (Fig. 1A). The implicated PPR genes corresponded to *AT1G66345*, *AT3G16010*, and *AT4G01400*. After molecular characterization of the corresponding mutants (see below), we named these genes Mitochondrial Intron Splicing Factor 26 (MISF26), Mitochondrial Intron Splicing Factor 68 (MISF68), and Mitochondrial Intron Splicing Factor 74 (MISF74), respectively. Two independent T-DNA lines were identified for each PPR gene. Each mutant pair exhibited very similar phenotypes, supporting that the observed growth alterations were due to the inactivation of the corresponding PPR genes. The lines *misf26-1* (SALK_008252), *misf26-2* (SAIL_363_F11), *misf68-2* (SALK_010785), *misf74-1* (SALK_054210), and *misf74-2* (GABI_191C05) contained T-DNA insertions within the PPR repeat coding regions of each corresponding gene, while the T-DNA of the *misf68-1* (SALK_062109) mutant is located upstream of the first PPR repeat coding sequence of *MISF68*. The genes *MISF26*, *MISF68*, and *MISF74* encode proteins that are predicted to comprise 11, 14, and 10 P-type PPR repeats, respectively, according to TPRpred (https://toolkit.tuebingen.mpg.de/#/tools/tprpred) and the PlantPPR database (http://ppr.plantenergy.uwa.edu.au/) (Fig. 1B). The accumulation of full-length transcripts derived from each PPR genes in the mutants could not be detected by RT-PCR analysis, strongly suggesting that the identified mutant lines corresponded to null mutants (see Supplementary Fig. S1). When grown under long day conditions (16 h light/8 h dark) in the greenhouse, *misf74-2*, *misf68*, and *misf26* showed severely delayed growth compared with Col-0 reference plants, and reached out about half the size of wild-type plants after 3 months of culture. Intriguingly, *misf74-1* plants exhibited a milder growth phenotype compared with *misf74-2* mutants (Fig. 1A).

### The AT4G01400, AT3G16010, and AT1G66345 PPR proteins are targeted to mitochondria *in vivo*

**Fig. 2.**
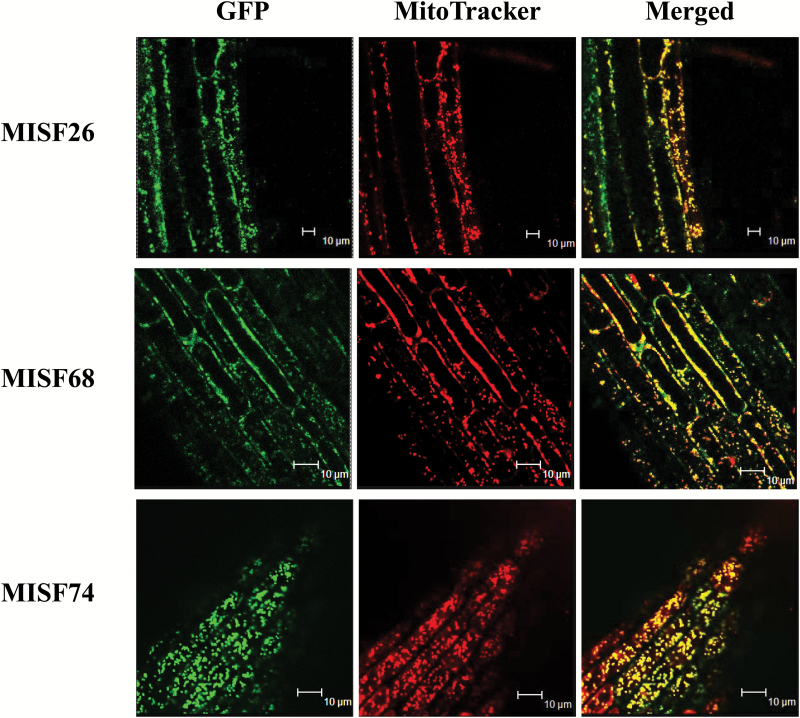
The MISF26, MISF68, and MISF74 genes encode mitochondria-targeted PPR proteins. Confocal microscope images showing the subcellular distribution of MISF: GFP translational fusions in root cells of young Arabidopsis transgenic plants. Prior to observation, roots were briefly soaked in MitoTracker Red to label mitochondria. The left panels show GFP fluorescence, the center show mitochondria labeled with the MitoTracker Red dye and the right panels present the merged signals.

The three identified PPR proteins were predicted to reside in mitochondria according to the Arabidopsis subcellular database SUBA (http://suba3.plantenergy.uwa.edu.au/). To verify their subcellular distribution, we generated GFP translational fusion constructs comprising the region encoding the putative mitochondrial transit peptide of each PPR protein and then transformed them into Arabidopsis Col-0 plants. Confocal microscopy was used to detect the GFP fluorescence in the roots of the generated transgenic plants. In all cases, we observed GFP signals that co-localized with MitoTracker red signals in mitochondria (Fig. 2), which confirmed the mitochondrial localization of the three PPR proteins *in vivo*.

### The three identified PPR genes are involved in intron splicing of distinct mitochondrially encoded transcripts

**Fig. 3.**
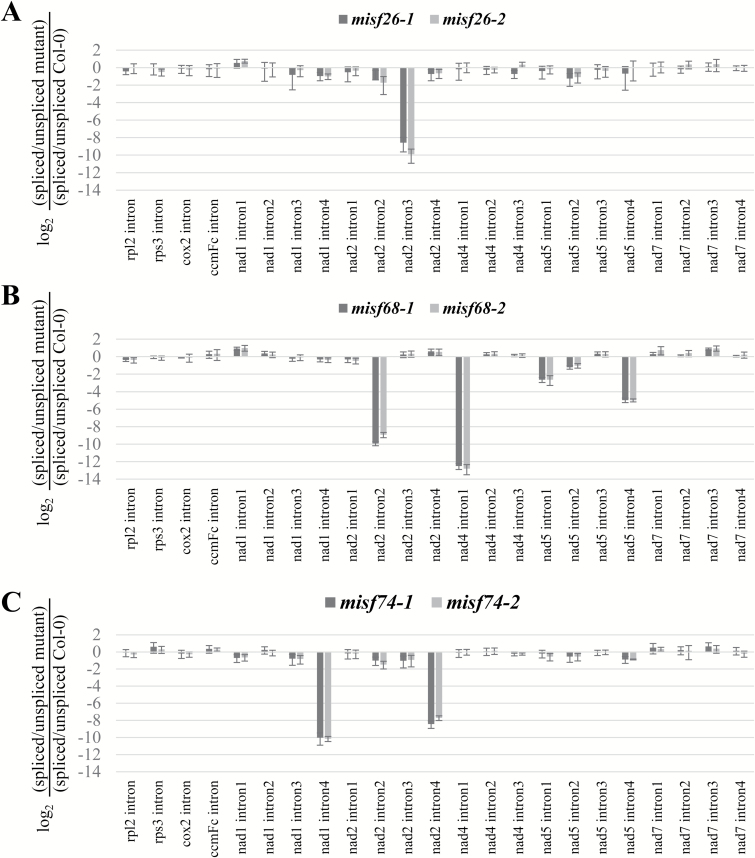
Splicing efficiencies of all mitochondrial introns were compared between the wild-type (Col-0) and the *misf* mutants. The bars depict the log_2_ ratio of spliced to unspliced forms for each intron in mutants as compared with the wild-type. Three technical replicates and two independent biological repeats were used for each genotype; standard errors are indicated. (A–C) show the results for the *misf26*, *misf68*, and *misf74* mutants respectively.

Previous studies have shown that PPR proteins are RNA-binding proteins and function in a wide range of mitochondrial and plastid RNA processing events (Barkan and Small, 2014; Hammani and Giegé, 2014). Our results indicated that the identified PPR proteins resided in mitochondria, which encouraged us to test whether they could play roles in mitochondrial RNA metabolism. To test this hypothesis, we first performed qRT-PCR to measure the steady state levels of mature mitochondrially encoded transcripts and determine the splicing efficiencies of all mitochondrial introns in each mutant line. No significant differences for most mature mitochondrial transcripts were found between the mutants and the wild-type (see Supplementary Fig. S2). However, a few specific regions of mRNAs encoding complex I respiratory subunits fail to accumulate to normal levels in the mutants. These concerned the *nad2* mRNA in *misf26*, *nad2* and *nad4* mRNAs in *misf68*, and *nad1* and *nad2* transcripts in *misf74* (Supplementary Fig. S2). To further specify the origin of these differences, we calculated the splicing efficiencies of all 23 introns in Arabidopsis mitochondria in the wild-type and mutant lines. As shown in Fig. 3, a single reduction in splicing efficiency was observed for *nad2* intron 3 (*cis*-intron) in *misf26* mutants (Fig. 3A). In contrast, several splicing defects were detected in *misf68* and *misf74* mutants (Fig. 3B, C). These concerned the *nad5* intron 4 (*cis*-intron), the *nad2* intron 2 (*trans*-intron) and the *nad4* intron 1 (*cis*-intron) in *misf68* plants (Fig. 3B). Regarding *misf74* plants, large reductions in the splicing of *nad1* intron 4 and *nad2* intron 4 (both are *cis*-introns) were detected (Fig. 3C).

**Fig. 4.**
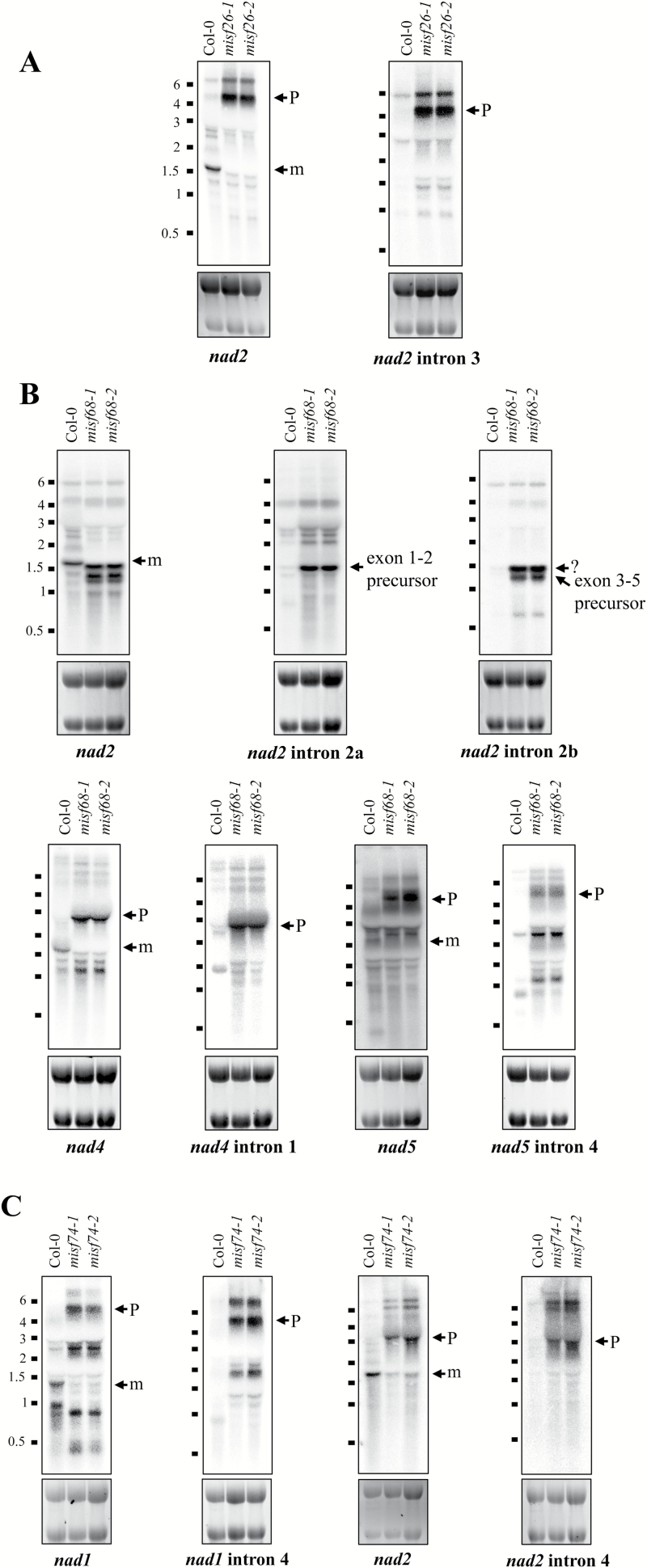
RNA gel blot analysis showing the accumulation pattern of affected complex I (*nad*) transcripts in the three *misf* mutants. Two probe sets were used in this assay: full-length cDNA probes to detect both mature and precursor transcripts and intron probes to specifically detect the affected precursor transcripts in each mutant. The blots were performed on total RNA isolated from *ppr* mutants and wild-type (Col-0) flowers and hybridized with the indicated probes. (A–C) show the corresponding results for *misf26*, *misf68*, and *misf74* as compared with the wild-type. *nad2* intron 2a and *nad2* intron 2b are specific probes detecting the 5′- and 3′-portions of *nad2* intron 2 (*trans*-intron). Ethidium bromide staining of ribosomal RNAs is shown below the blots and serves as a loading control. RNA marker sizes are indicated. m, mature mRNAs; p, precursor transcript.

RNA gel blots were then carried out to confirm the results obtained from qRT-PCR analysis. Two kinds of probe were used for each affected transcript in this assay, the first one detecting mRNA exons of genes for which splicing defects were revealed and the second one specific to the impacted introns. The results showed that no mature transcripts for the impacted genes could be detected in any of the *misf* mutants, except for *nad2* in *misf74* plants in which trace amount of mature transcripts could be found. Accordingly, precursor transcripts comprising the introns whose splicing is reduced overaccumulated in all mutant lines (Fig. 4).

Altogether, these results allowed us to conclude that the three newly identified Arabidopsis PPR genes are involved in splicing of different mitochondrial group II introns. MISF74 and MISF68 are required for splicing of two and three introns each, whereas MSIF26 is essential for a single one.

### Complex I biogenesis is strongly affected in *misf26*, *misf68*, and *misf74* mutants

**Fig. 5.**
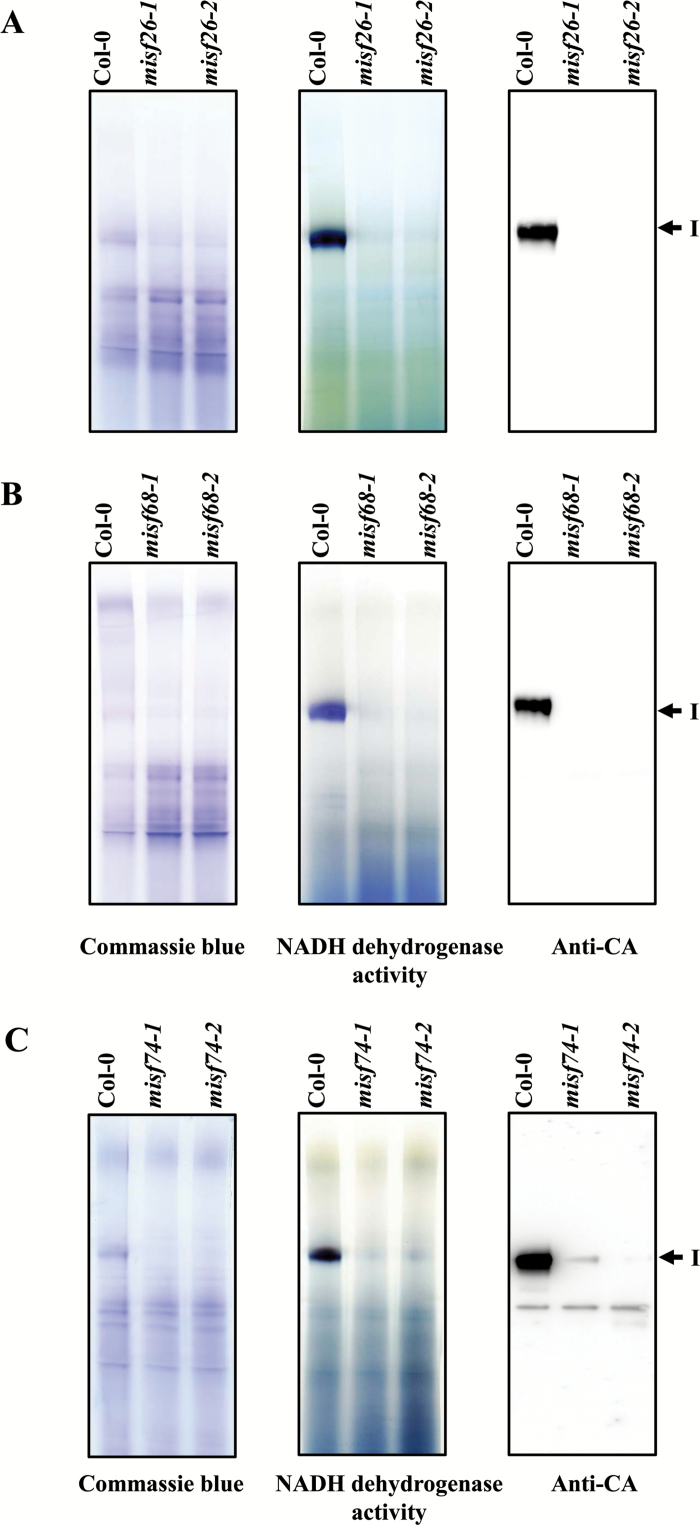
Complex I biogenesis is strongly affected in *misf* mutants. Crude mitochondrial extracts were prepared from wild-type and mutant plants, separated on BN-PAGE gels and stained with Coomassie blue (left panel). In-gel staining revealing the NADH dehydrogenase activity of complex I is also present in the center. Following migration, BN-PAGE gels were transferred to membranes and blots were hybridized with antibodies to mitochondrial CA subunit (right panel). The holocomplex I is indicated by an arrowhead. (A–C) show the corresponding results for *misf26*, *misf68*, and *misf74* mutants, respectively.

The mitochondrial *nad* genes encode essential subunits of respiratory complex I (NADH: ubiquinone oxidoreductase). The various splicing defects detected in the different *misf* mutants led us to consider that the corresponding plants may be affected in the production of complex I. We thus used BN-PAGE to separate the different mitochondrial respiratory complexes and estimate the abundance of complex I in wild-type plants and the three *misf* mutants. Crude mitochondrial extracts were prepared from both types of plants and solubilized in 1% *n*-dodecyl β-D-maltoside. Following gel migration, the abundance of respiratory complex I was visualized either by gel activity staining or by western blot analysis using antibodies recognizing the γ-carbonic anhydrases (CA) associated with complex I. These analyses revealed that the accumulation levels of complex I were significantly reduced in all *misf* mutants compared with the wild-type (Fig. 5). Intriguingly, a slight amount of complex I could be detected in the *misf74-1* mutant but not in the *misf74-2* mutant (Fig. 5C), which correlated with the milder growth alteration of *misf74-1* compared with *misf74-2* plants (Fig. 1).

To further dissect the respiratory re-orientations resulting from the lack of complex I in the *misf* mutants, we examined the expression levels of the alternative oxidase (AOX) and NADH dehydrogenase (*NDA*, *NDB*, and *NDC*) genes by qRT-PCR. This analysis revealed a strong overaccumulation of *AOX1A*, *NDA1*, and *NDB4* transcripts in most *misf* mutants compared with the wild-type. In contrast, the abundance of *AOX2* mRNA was largely reduced in the *misf* mutants (see Supplementary Fig. S3).

Overall, our observations indicated that *misf26*, *misf68*, and *misf74* mutants represent complex I deficient plants and that the alternative respiratory pathway is consequently activated in these lines. The loss of proper splicing for distinct *nad* transcripts in these mutants strongly suggests that these are responsible for the complex I deficiencies detected in these plants.

## Discussion

### MISF26, MISF68, and MISF74 are required for the splicing of various mitochondrial introns and affect complex I assembly and activity in Arabidopsis

The expression of mitochondrial transcripts in plants requires the activity of a plethora of nuclear-encoded *trans*-factors among which PPR proteins are by far the most represented class of proteins. This study reveals the function of three new mitochondria-targeted PPR proteins and shows that they are essential for the splicing of six separate mitochondrial introns. To reach such conclusions, we first observed that GFP translational fusions comprising the N-terminal regions of each of these PPR proteins localized in Arabidopsis mitochondria, strongly supporting the mitochondrial targeting of these proteins. The analysis of mitochondrial mRNAs in T-DNA insertion mutants clearly indicated that MISF26, MISF68, and MISF74 are indispensable for the splicing of one, three, and two mitochondrial introns, respectively (Figs 3, 4). The MISF26 PPR protein is crucial for the splicing of the third intron of the *nad2* pre-mRNA. MISF68 was found to be essential for the removal of *nad2* intron 2, *nad4* intron 1, and *nad5* intron 4, although the importance of MISF68 for this last intron appears to be weaker than for the other two. Regarding MISF74, we found that this PPR protein facilitates the splicing of both *nad1* intron 4 and *nad2* intron 4. These different splicing defects therefore impact the production of various mitochondrially encoded complex I subunits and accordingly all *misf* mutants show slow growth phenotypes that are typical of complex I mutants and that correlate with a very strong decrease of this respiratory complex (Figs 1A, 5). To compensate for the strong reduction in complex I accumulation, we found a strong increase in the steady state levels of mRNAs encoding alternative NADH dehydrogenases, notably NDA1 and NDB4, in the different *misf* mutants (see Supplementary Fig. S3).

### MISF68 and MISF74 are multi-functional PPR proteins

Interestingly, no overlap was found among the intron targets of the three MISF proteins. The *cis*- and *trans*- nature of mitochondrial introns does not explain this lack of overlap, as the MISF68 protein facilitates the splicing of both types of introns. Two functional categories of *trans*-factors seem to play roles in intron splicing in plant mitochondria. Factors like maturases, RNA helicases or CRM (chloroplast RNA splicing and ribosome maturation) proteins are necessary for the removal of a large number of group II mitochondrial introns and can be considered as general splicing factors. They may be part of the core of a mitochondrial spliceosomal complex (if such complex exists) and are thereby required for the elimination of, virtually, all mitochondrial introns. The second category concerns factors like the PPR proteins that, in most cases, are necessary for the splicing of a single intron (see Supplementary Fig. S4). This second class of *trans*-factors seem to act as accessory factors aiding the recruitment of the mitochondrial splicing machinery on specific introns and/or by somehow improving the activity of this machinery on specific intron targets. The incorporation of intron-specific factors in the mitochondrial splicing machinery could result from sequence or structural changes in certain introns, creating mono-specific splicing complexes. Interestingly, our results indicate that some of these accessory factors can facilitate the splicing of more than one intron, as MISF74 is required for the elimination of two introns and three introns necessitate MISF68 for their splicing. The multi-functionality of PPR proteins in organellar RNA metabolism has been described in a few cases, notably for PPR engaged in RNA editing, but was rarely found for PPR proteins involved in splicing (de Longevialle *et al.*, 2008; Khrouchtchova *et al.*, 2012; Barkan and Small, 2014; Hammani and Giegé, 2014; Wang *et al.*, 2017*b*). MISF68 and MISF74 represent two of the first counterexamples found in plant mitochondrion. The molecular functions of PPR proteins in splicing are still unclear but it has been suggested that they may help intron folding and stabilize their structure in a catalytically active form (Beick *et al.*, 2008; Williams-Carrier *et al.*, 2008; Solem *et al.*, 2009; Barkan and Small, 2014; Aryamanesh *et al.*, 2017). The multi-functionality of MISF68 and MISF74 may result from the recognition of the same functional domain shared by their intron targets, implying that they could play the same role in the splicing of the introns they facilitate. Some functional differences can be anticipated, though, as the lack of MISF68 does not impact the splicing efficiency of its different intron targets to the same extent (Fig. 3B). The multiplicity of roles that PPR proteins could play in splicing is also suggested by the fact that several of them are necessary for the removal of the same mitochondrial intron, such as ABO5 and MISF26 in Arabidopsis or Dek37 and EMP10 in maize (Liu *et al.*, 2010; Cai *et al.*, 2017; Dai *et al.*, 2018). Finding the RNA biding sites of these PPR within their intron targets is certainly an important prerequisite for understanding the function of these proteins in splicing. It has been recently established that PPR proteins may recognize their RNA targets via a one PPR motif—one nucleotide rule, and that combinations of amino acids at positions 5 and 35 of each repeat are determinants for RNA base selection (Barkan *et al.*, 2012; Takenaka *et al.*, 2013*a*; Yagi *et al.*, 2013; Kindgren *et al.*, 2015; Shen *et al.*, 2016). To predict their potential RNA binding sites, we applied the PPR-RNA recognition code to the three MISF proteins. Several candidate sites within most intron targets could be identified, except for MISF68 in *nad5* intron 4 (Supplementary Fig. S5). However, the identified sites ranked rather low in the prediction lists for each protein, likely because several amino acid combinations at positions 5 and 35 of MISF PPR repeats are not filled by the PPR code. Advanced biochemical analyses such as RIP-Seq or similar kinds of approaches are thus necessary to identify the binding sites of these newly identified PPR proteins.

### The three MISF proteins show different conservation levels in plants

Recent analyses have indicated that most PPR genes in plants appeared before the divergence of monocot and dicot species and that potential orthologs of Arabidopsis PPR proteins can be quite easily identified in other plant species (O’Toole *et al.*, 2008). We compared protein similarities and identities between flowering plant orthologs of the three MISF proteins and found, surprisingly, that the levels of sequence identity varied quite extensively between them. With more than 80% of sequence identity between othologs, MISF68 appeared to be the most conserved factor among the three splicing proteins. The level of sequence identity between MISF74 homologs is around 74% whereas it is only around 60% for MISF26 closely related homologs (see Supplementary Fig. S6). Specific functional constraints may have limited the sequence diversification of MISF68 and MISF74 compared with MISF26, which could result from the fact that MISF68 and MISF74 facilitate the splicing of multiple introns. Because of the necessity to interact with several RNA targets, PPR proteins carrying multifarious activities may be effectively less incline to sequence changes throughout evolution. The high sequence conservation between MISF potential orthologs (Supplementary Fig. S6) suggested similar conservation levels for their *in vivo* RNA binding sites. Multiple sequence alignments were thus performed to see if any of the predicted target sites (Supplementary Fig. S5) could show higher sequence conservation compared with others. Certain sites show perfect or almost perfect sequence conservation and thus represent serious target site candidates of MISF proteins (Supplementary Fig. S7). Major genetic and biochemical advances are required before we understand how PPR proteins assist group II intron splicing and how that influences their sequence diversification. Taken together, MISF68 and MISF74 might serve as novel models to understand the multiple functions of PPR proteins, and their molecular deciphering will provide new insight into the mechanisms of intron splicing in plant mitochondria.

## Supplementary data

Supplementary data are available at *JXB* online.

Fig. S1. RT-PCR analysis of each MISF transcript in wild-type and *misf* mutants.

Fig. S2. Various mature mitochondrial transcripts underaccumulate in *misf* mutants.

Fig. S3. Quantitative RT-PCR measuring the steady-state levels of mRNAs encoding alternative oxidases (AOX) and NADH-dehydrogenases (NDA, NDB and NDC).

Fig. S4. Listing of known nucleus-encoded proteinaceous factors involved in the splicing of mitochondrial group II introns in Arabidopsis.

Fig. S5. Predictions of MISF protein binding sites according to the PPR code.

Fig. S6. Multiple sequence alignments of MISF protein homologs from a representative selection of dicot and monocot plant species.

Fig. S7. Multiple sequence alignments analyzing the conservation of the predicted RNA targets of each MISF protein.

Table S1. Oligonucleotides used in this study.

Supplementary Figures S1-S7 and Table S1
