# Supplementary material for: Three new pentatricopeptide repeat proteins facilitate the splicing of mitochondrial transcripts and complex I biogenesis in Arabidopsis
Source: J Exp Bot. 2018 Jul 25;69(21):5131–40. doi: 10.1093/jxb/ery275 (PMC6184586; doi:10.1093/jxb/ery275)
Supplement: Supplementary Figures S1-S7 and Table S1 [file ery275_suppl_supplementary_figures_and_table.pdf]

Name	Sequence 5'-3'	Experiment
GWPPR-P26-7	CAAAAAAGCAGGCTAAAATGGCGTCAGCTTTGCGCCG	GFP expression
GWPPR-P26-8	CAAGAAAGCTGGGTCAGAAGATGAGATCTCATAAG	
GWPPR-P68-5	CAAAAAAGCAGGCTAAAATGATGTATGTATCTGCTCG	
GWPPR-P68-6	CAAGAAAGCTGGGTCAACGCTAACATATGTGTTAC	
GWPPR-P74-3	CAAAAAAGCAGGCTAAAATGATTCGCCGGCCGATCTA	
GWPPR-P74-6	CAAGAAAGCTGGGTCAGAATGGCGGAAATTGGGCTG	
PPR-P26-3	CGAACGAGATTACGATTCGG	Genotyping
PPR-P26-1	TTTGGTGGAATGGAAGTTGAC	
PPR-P26-2	TGTTTAGGGTTTTGGAAGAGATG	
PPR-P68-1	TGCAATTTTGGAATAGTTCGG	
PPR-P68-2	CCGAGTTGTAAGTGCTCGAAG	
PPR-P68-3	TTCAGTTTTTCAAATGGGCTG	
PPR-P68-4	GCTCATTCGCTGCTTCATATC	
PPR-P74-1	GCATGCCAGAATACGGTTATG	
PPR-P74-2	ATCCACATCAGGGACAACATC	
PPR-P74-4	TAACTGAGATACCGCACCACC	
PPR-P74-7	AGCTTTTCAGGATCAGGAGGA	
LB-Gabi1	CCCATTTGGACGTGAATGTAGACAC	
LB-Salk2	GCTTTCTTCCCTTCCTTTCTC	
LB3-Sail	TAGCATCTGAATTTCATAACCAATCTCGATACAC	
RT-P26-1	TGGAGGATTTACGAATGTGCGA	RT-PCR
RT-P26-2	TTCATCGGGCACAAACCCTT	
RT-PR68-1	TCAGTGCGGATGTTGGATGA	
RT-PR68-2	CACTCGACCCACTTTGCCTA	
RT-P74-1	CGCAATCGGATCCTCTCCTC	
RT-P74-2	ACCTATCAGGCACGAATCCTT	
nad1e7	CATCACCCTAGCAAGCCTAAC	northern blot, nad1 probe
nad1e8	TAAGGAAGCCATTGAAAGGTG	
nad1 int4-1	ACGGGAAAGTTGACTCCTGG	northern blot, nad1 intron 4 probe
nad1 int4-2	AGGGCTTCCCTCAGTTCAGT	
nad2e1	GCAGAATTCGTTCGGATC	northern blot, nad2 probe
nad2e8	TATGAACTGAGTGCCATTTGA	
nad2 int2a-1	ATGACGAAAGGAGAGTCGGC	northern blot, nad2 intron 2a probe
nad2 int2a-2	TTCTGGCCCCTGTGAACATC	
nad2 int2b-1	CCAACGGGGAATAGAAGCGA	northern blot, nad2 intron 2b probe
nad2 int2b-2	ATACGGCTCGCACAAAGACT	
nad2 int3-1	TTAGTCAATGGGGCAGCAGG	northern blot, nad2 intron 3 probe
nad2 int3-2	TCACACGAAAGGGAACGAGG	
nad2 int4-1	GGGAATGCATGCTACGAAAGA	northern blot, nad2 intron 4 probe
nad2 int4-2	TTTCCGCCCCCAATGAGATG	
nad4F	ATTCTATGTTTTTCCCGAAAGC	northern blot, nad4 probe
nad4R	TGAAATTTGCCATGTTGCAC	
nad4 int1-1	CCCGCCCAATCCAACTAAGA	northern blot, nad4 intron 1 probe
nad4 int1-2	AGACCTGGACGATTATCCCT	
nad5F	AACATTGCAAAGGCATAATGA	northern blot, nad5 probe
nad5R	CCATGGATCTCATCGGAAAT	
nad5 intron4-1	TCCCTATGGACAAGGGGACA	northern blot, nad5 intron 4 probe
nad5 intron4-2	ACTCTTCATTCTCCCGTGCT	

Supplementary Table S1: Oligonucleotides used in this study.

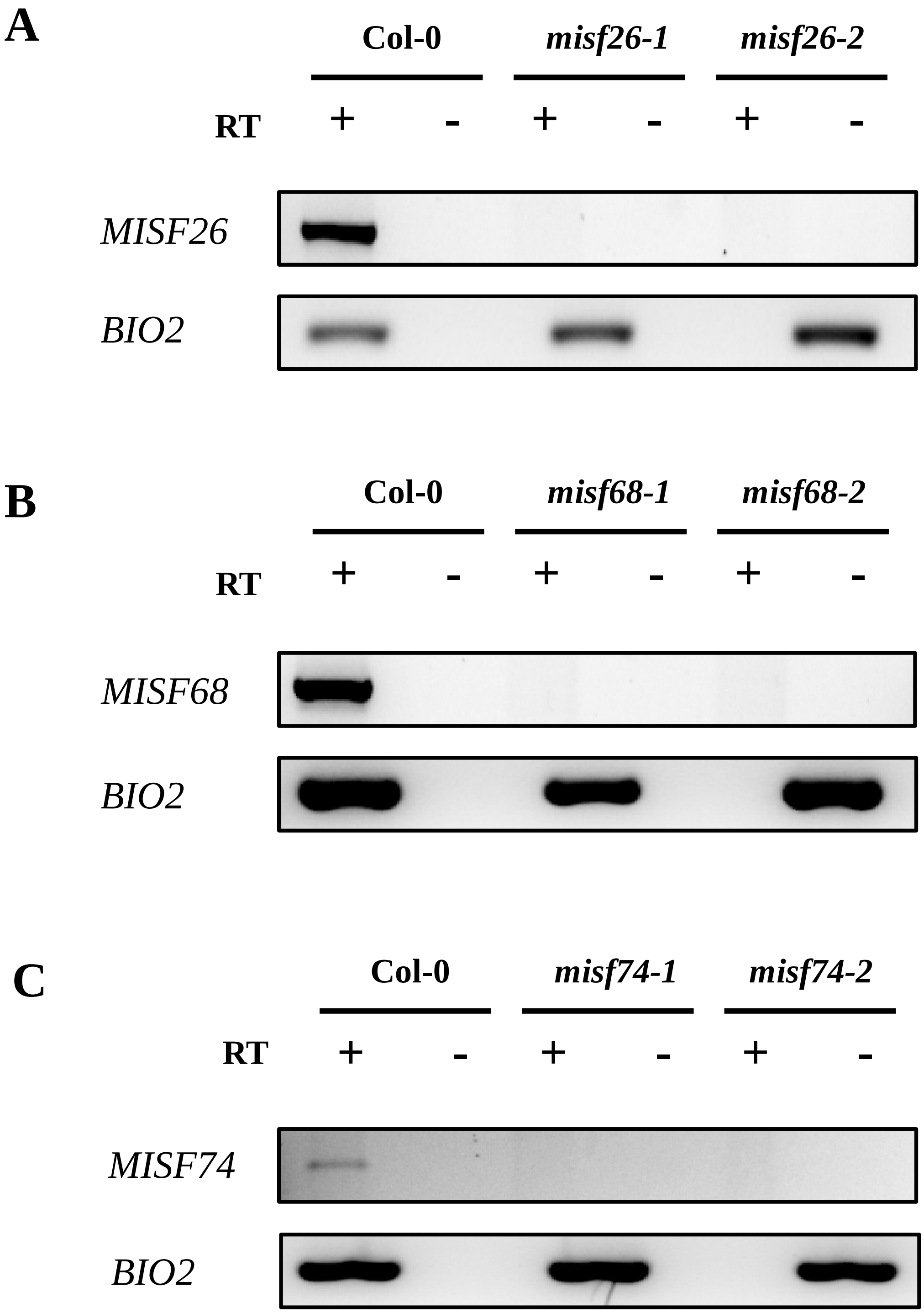

Supplementary Figure S1: RT-PCR analysis of each *MISF* transcript in wild-type and *misf* mutants. cDNA prepared from both wild-type and *misf* plants were PCR amplified using primers located on both sides of the T-DNA insertion sites corresponding to each *misf* mutation, and the resulting amplification products were size fractionated on agarose gel. The amplification of *BIO2* cDNA was done separately to control the efficiency of the RT reaction. Reverse transcriptase was either added (RT+) or omitted (RT-) from the initial RT reaction.

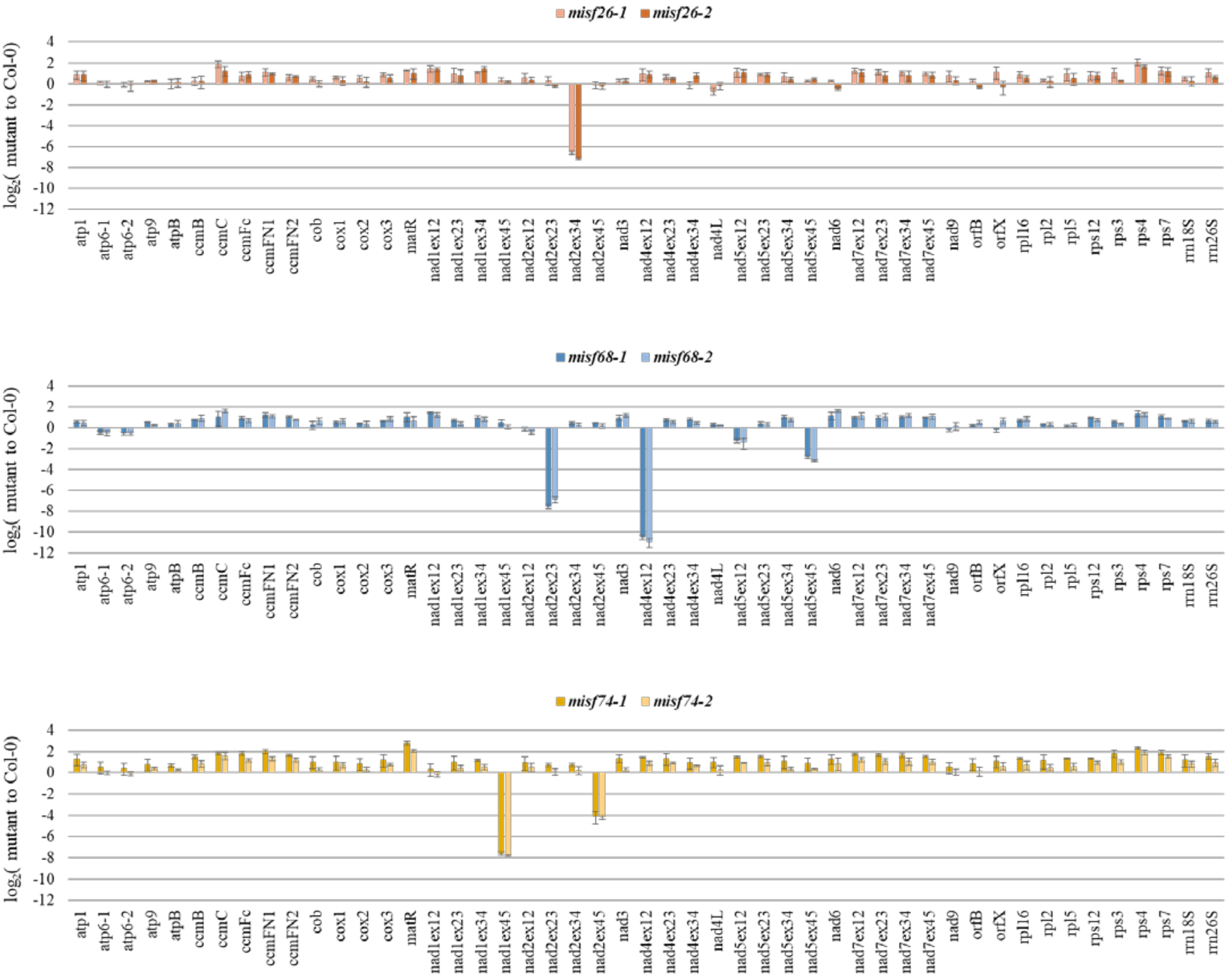

Supplementary Figure S2: Various mature mitochondrial transcripts under-accumulate in *misf* mutants. The steady state levels of mature mitochondrial mRNAs measured by quantitative RT-PCR in Col-0 and *misf* mutant plants. The histograms show the $\log_2$ ratio of mature transcripts in mutants to the wild-type (Col-0). A single PCR was considered for mRNAs carrying no introns, whereas the accumulation of individual exons was analyzed for intron-containing transcripts. Three technical replicates of two independent biological repeats were used for each genotype; standard errors are indicated. The data were normalized to the nuclear 18S rRNA gene.

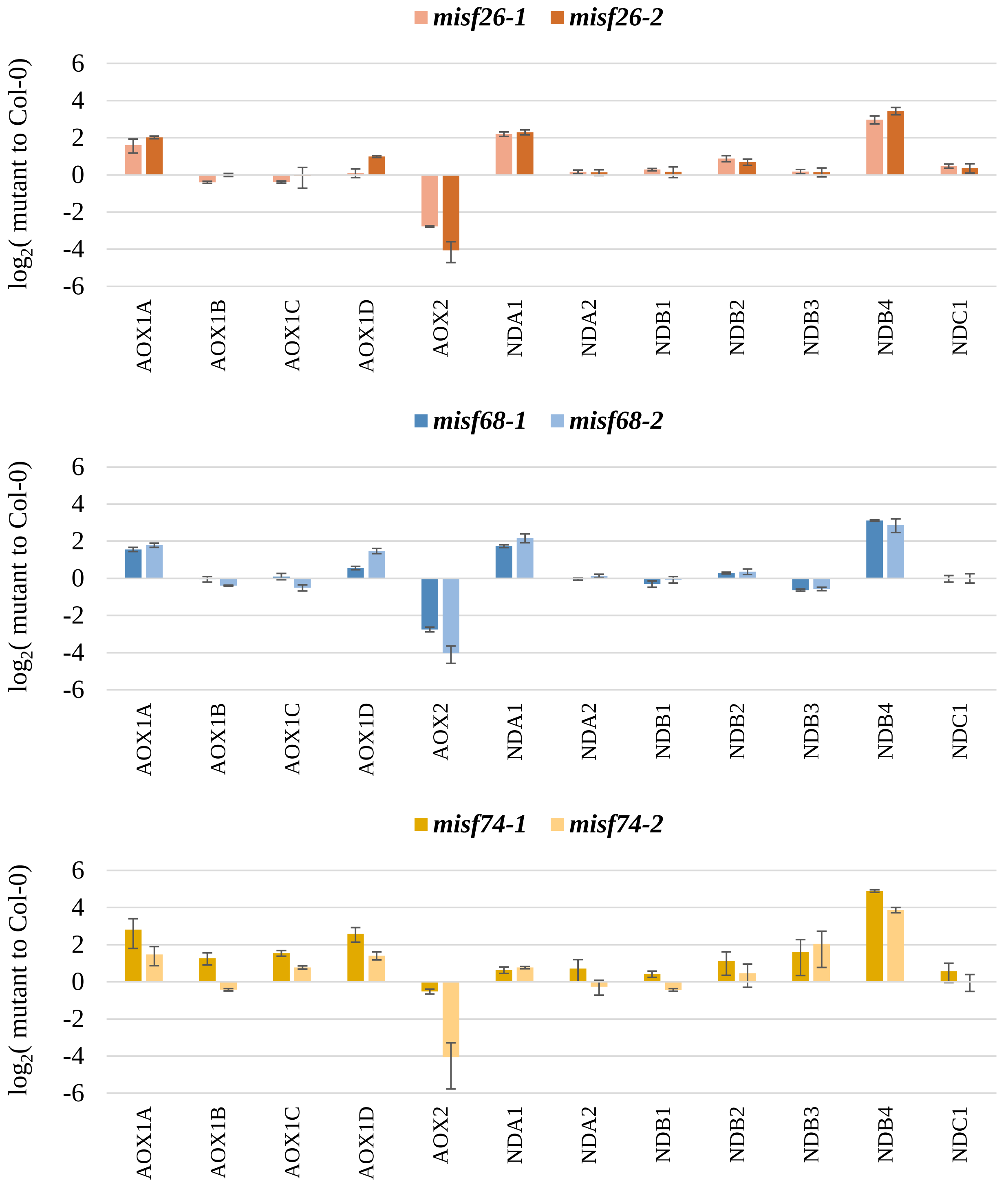

Supplementary Figure S3: Quantitative RT-PCR measuring the steady-state levels of mRNAs encoding alternative oxidases (AOX) and NADH-dehydrogenases (NDA, NDB and NDC). Three technical replicates of two independent biological repeats were used for each genotype; standard errors are indicated.

	Family	PPR										Maturase			Helicase		CRM	PORR	RCC1-like	mTERF	RAD52-like
	splicing factors	ABO5	ABO8	BIR6	MISF26	MISF68	MISF74	OTP43	OTP439	SLO3	TANG2	nMAT1	nMAT2	nMAT4	PMH2	ABO6	mCSF1	WF9	RUG3	mTERF15	ODB1
group II introns	***nad1 intron 1***							■				■		■							
	nad1 intron 2												■		■	■	■				■
	nad1 intron 3												■	■	■	■	■				
	nad1 intron 4						■							■		■					
	nad2 intron 1											■	■		■		■				■
	nad2 intron 2					■									■		■				
	nad2 intron 3	■			■											■	■		■	■	
	nad2 intron 4						■						■		■	■	■				
	nad4 intron 1					■										■					
	nad4 intron 2											■	■		■	■					
	nad4 intron 3		■												■	■					
	nad5 intron 1												■		■	■	■				
	nad5 intron 2								■				■		■	■	■				
	nad5 intron 3										■		■		■	■	■				
	nad5 intron 4					■															
	nad7 intron 1			■									■								
	nad7 intron 2									■			■				■				
	nad7 intron 3														■						
	nad7 intron 4																				
	ccmFC intron																	■			
	cox2 intron												■		■		■				
	rpl2 intron														■			■			
	rps3 intron												■		■		■				

Supplementary Figure S4: Listing of known nucleus-encoded proteinaceous factors involved in the splicing of mitochondrial group Ⅱ introns in Arabidopsis. Different nucleus-encoded protein factors assisting mitochondrial intron splicing has been identified *via* genetic screens in Arabidopsis so far. Bold black indicate trans-spliced introns. Introns whose splicing is affected by the indicated trans-factors are indicated by orange boxes. Corresponding references are cited in the introduction.

A

	aa 5	D	N	R	T	S	T	N	N	S	R	D
	aa 35	S	N	S	D	N	Y	S	D	G	N	R
	Prediction	N	C/U	N	G	A	N	C	U/C	N	N	N
												p-value
***nad2* intron 3**	BS1	G	C	A	G	A	C	C	U	U	U	C	4.84E-04
	BS2	G	C	G	G	A	C	C	U	U	U	U	4.84E-04
	BS3	G	C	G	G	A	G	C	C	C	G	U	8.55E-04

B

	aa 5	M	S	N	S	T	T	N	N	S	C	A	N	N	N	
	aa 35	S	T	D	T	T	D	T	S	C	S	D	D	D	D	
	Prediction	N	G/U	U/C	G/U	C	G	C/U	C	N	C/U	U/G	U/C	U/C	U/C	*p*-value
***nad2* intron 2**	BS1	C	U	U	G	G	G	C	U	G	U	G	U	C	U	1.35E-04
	BS2	C	U	U	U	C	U	U	C	U	U	U	C	U	U	4.18E-04
	BS3	C	G	U	C	C	G	C	C	U	A	U	C	U	U	5.53E-04
	BS4	C	U	U	C	C	G	G	C	U	G	G	C	U	U	7.44E-04
	BS5	C	U	U	U	C	A	U	U	U	G	G	C	U	U	7.55E-04
	BS6	C	C	U	G	C	A	C	C	U	U	A	U	U	U	8.48E-04
***nad4* intron 1**	BS1	G	U	U	G	C	G	U	U	C	G	G	U	U	C	2.19E-04
	BS2	U	U	U	U	C	A	G	U	A	U	G	U	C	U	7.97E-04

C

	aa 5	T	L	T	R	N	K	T	N	R	N	
	aa 35	H	T	P	N	D	D	D	N	H	H	
	Prediction	A/G	U	A	N	U/C	G/C	G	C/U	N	C/U	*p*-value
***nad1* intron 4**	BS1	G	U	A	G	U	C	G	G	C	C	1.41E-04
***nad2* intron 4**	BS1	A	U	C	U	U	C	G	C	C	C	6.82E-04

Supplementary Figure S5: Predictions of potential RNA target sites for each MISF protein according to the PPR code. The amino acids at positions 5 and 35 were extracted from each MISF PPR repeat and are listed from N- to C-terminus. These combinations were used to identify potential RNA recognition sites of each MISF protein within their intron targets according to the PPR code. The RNA targets within introns were shown in different background colors, and nucleotides that match the predicted RNA recognition code are marked in red. The *P-values* were determined using the FIMO program as previously described (Wang et al., 2017a). (A), (B) and (C) show the results for MISF26, MISF68 and MISF74, respectively.

A

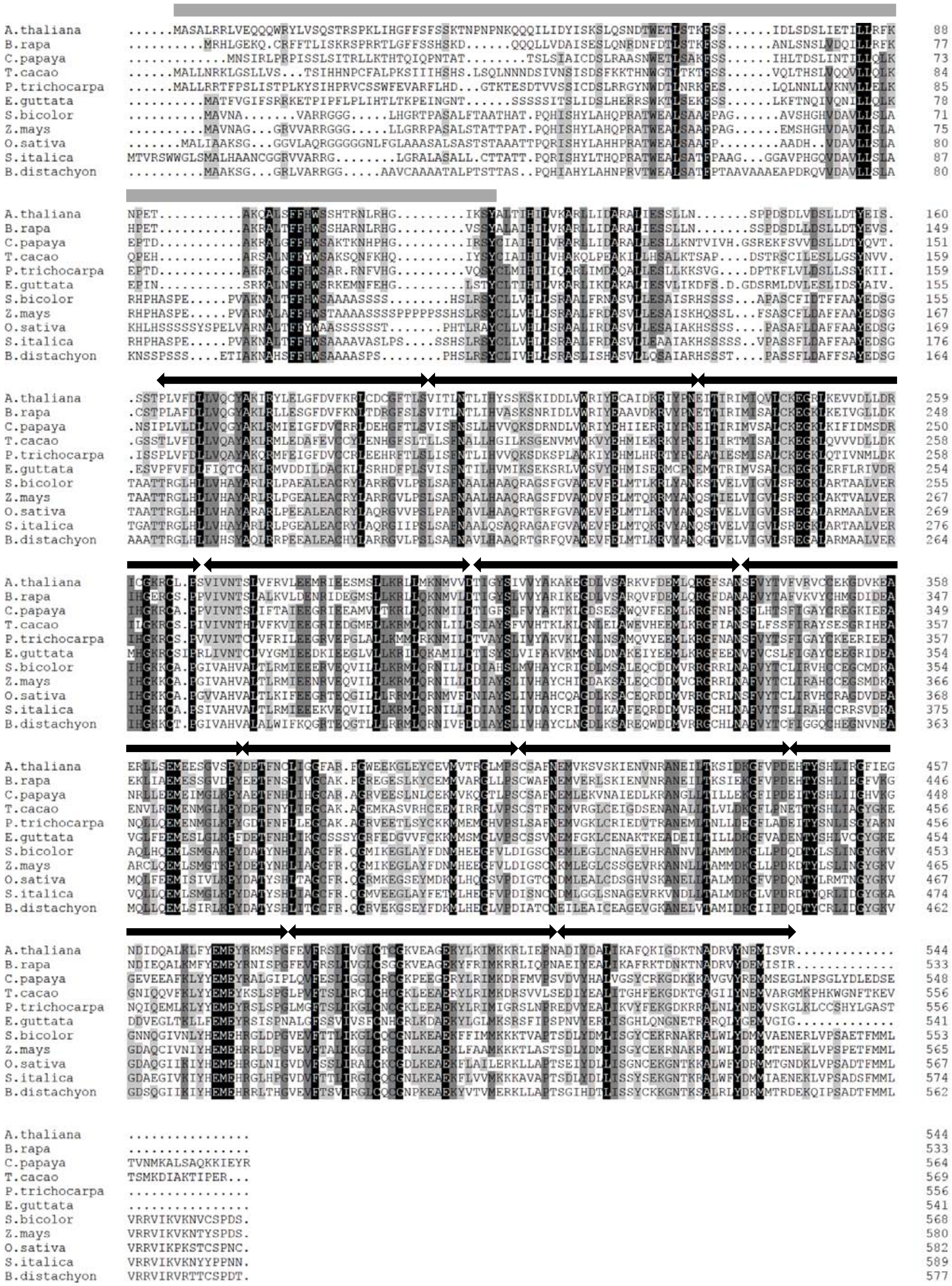
A.thaliana
B.rapa
C.papaya
T.cacao
P.trichocarpa
E.guttata
S.bicolor
Z.mays
O.sativa
S.italica
B.distachyon

B

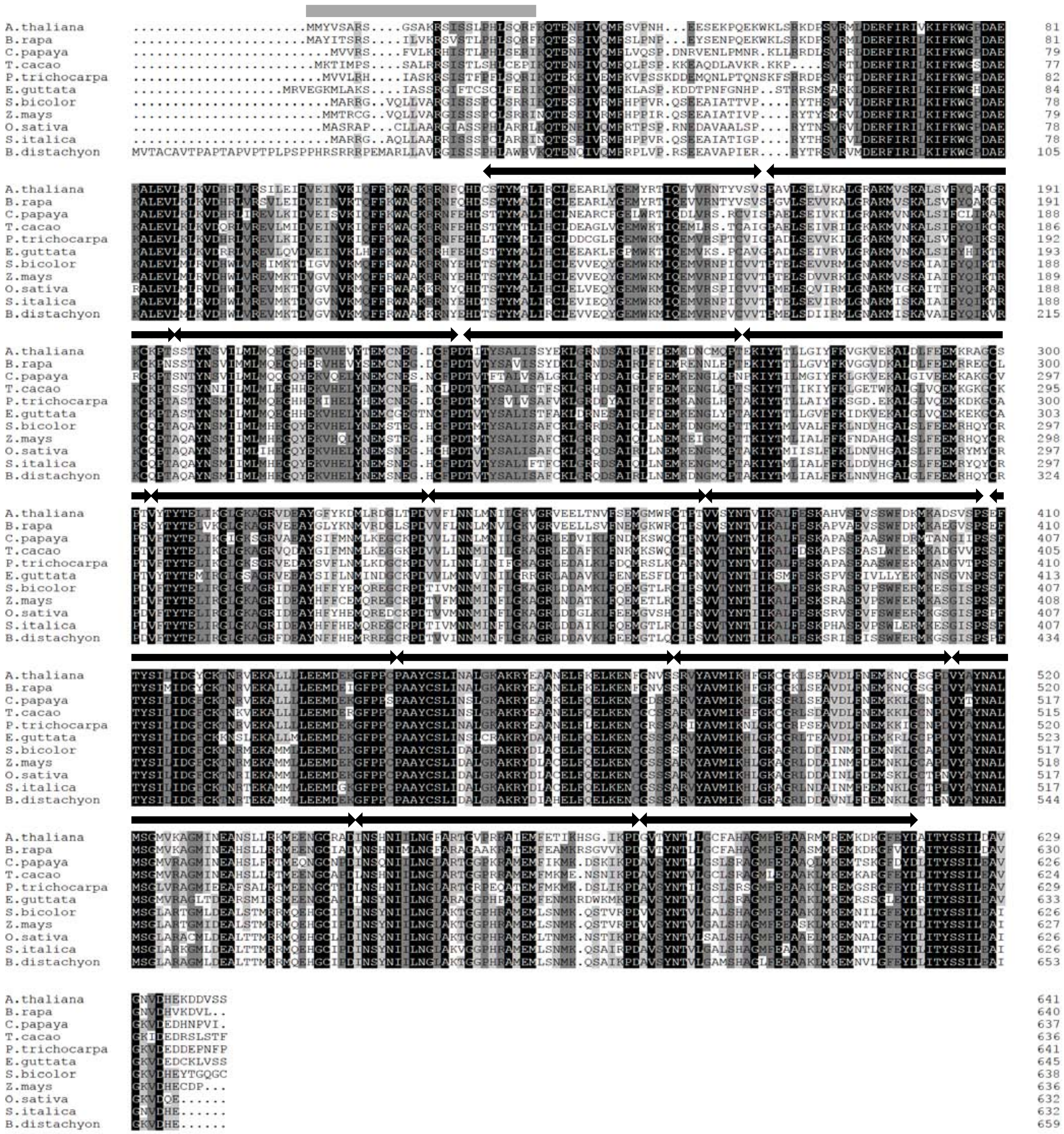
A.thaliana
B.rapa
C.papaya
T.cacao
P.trichocarpa
E.guttata
S.bicolor
Z.mays
O.sativa
S.italica
B.distachyon

C

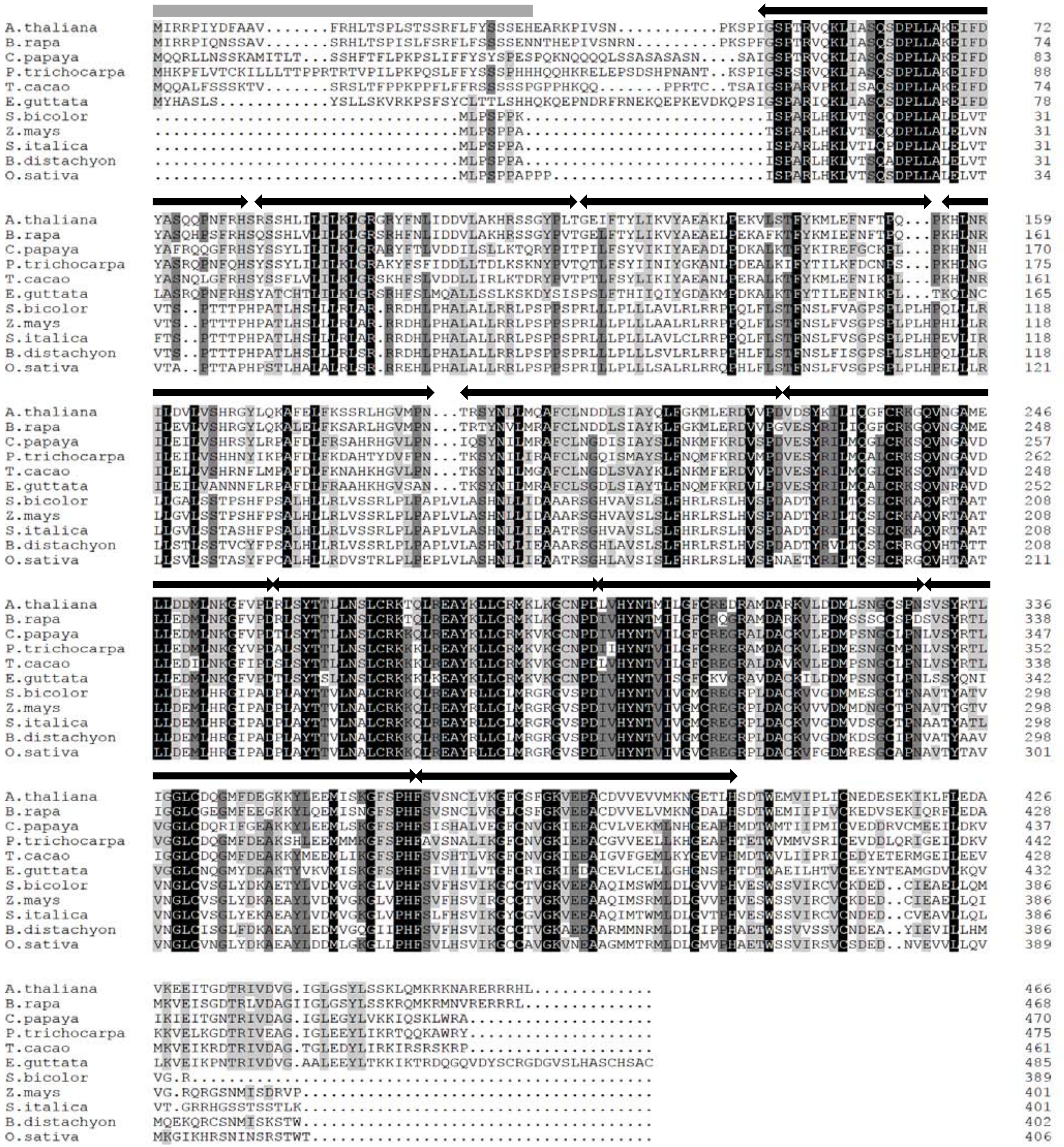

Supplementary Figure S6: Multiple sequence alignments of MISF protein homologs from a selection of dicot and monocot plant species. Amino acid sequence alignments of three MISF proteins from a representative selection of dicot (*Arabidopsis thaliana*, *Brassica rapa*, *Carica papaya*, *Theobroma cacao*, *Populus trichocarpa*, *Erythranthe guttata*) and monocot (*Oryza sativa*, *Zea mays*, *Sorghum bicolor*, *Setaria italic*, *Brachypodium distachyon*) plant species. Identical and conserved amino acids were shaded in black and grey respectively. PPR motifs are indicated by double arrow lines. Grey bars represent protein regions corresponding to mitochondrial targeting sequences, according to TargetP predictions. (A), (B) and (C) show the corresponding results from MISF26, MISF68 and MISF74 alignments.

A

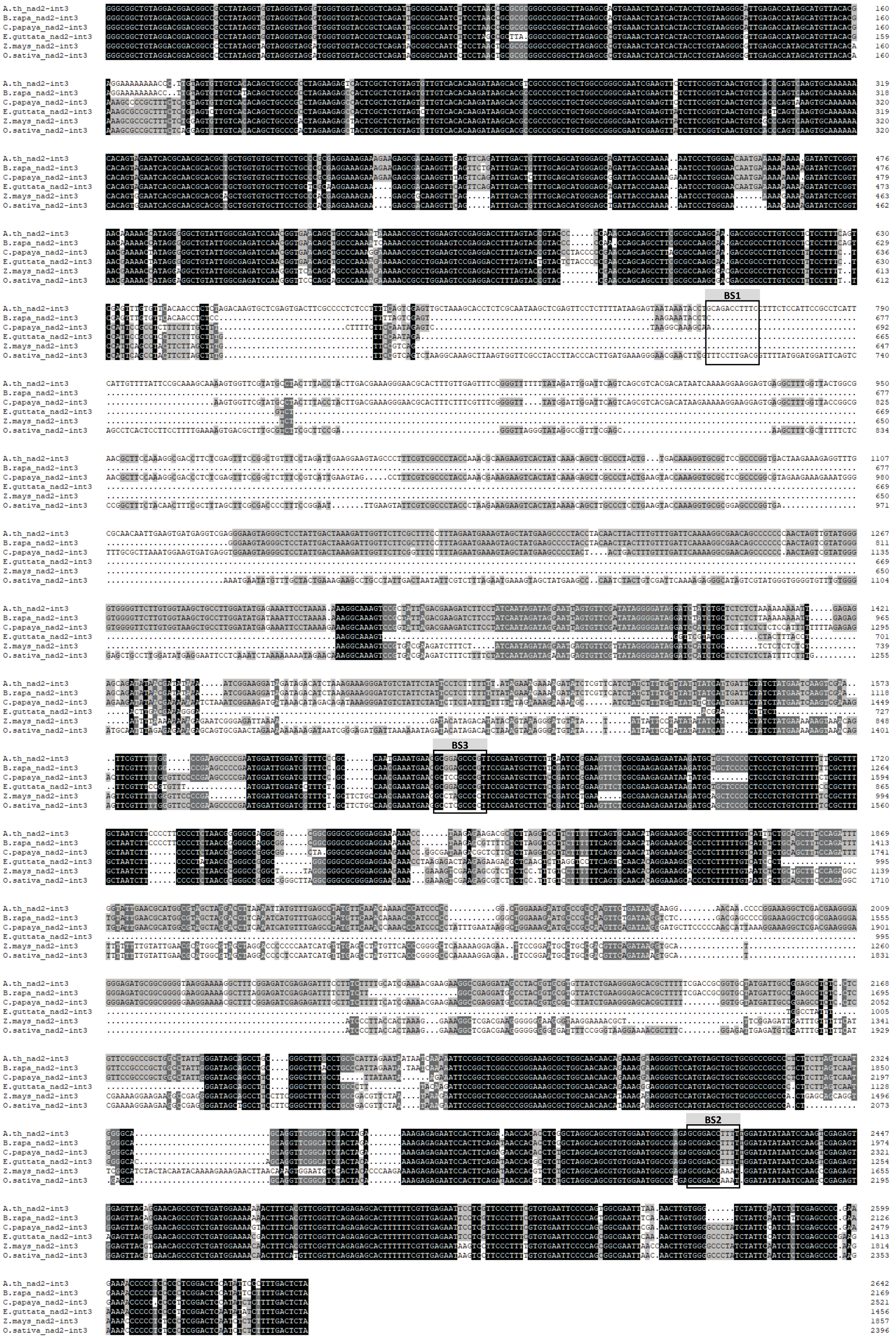
A.th_nad2-int3
B.rapa_nad2-int3
C.papaya_nad2-int3
E.guttata_nad2-int3
Z.mays_nad2-int3
O.sativa_nad2-int3
BS1
BS3
BS2

B

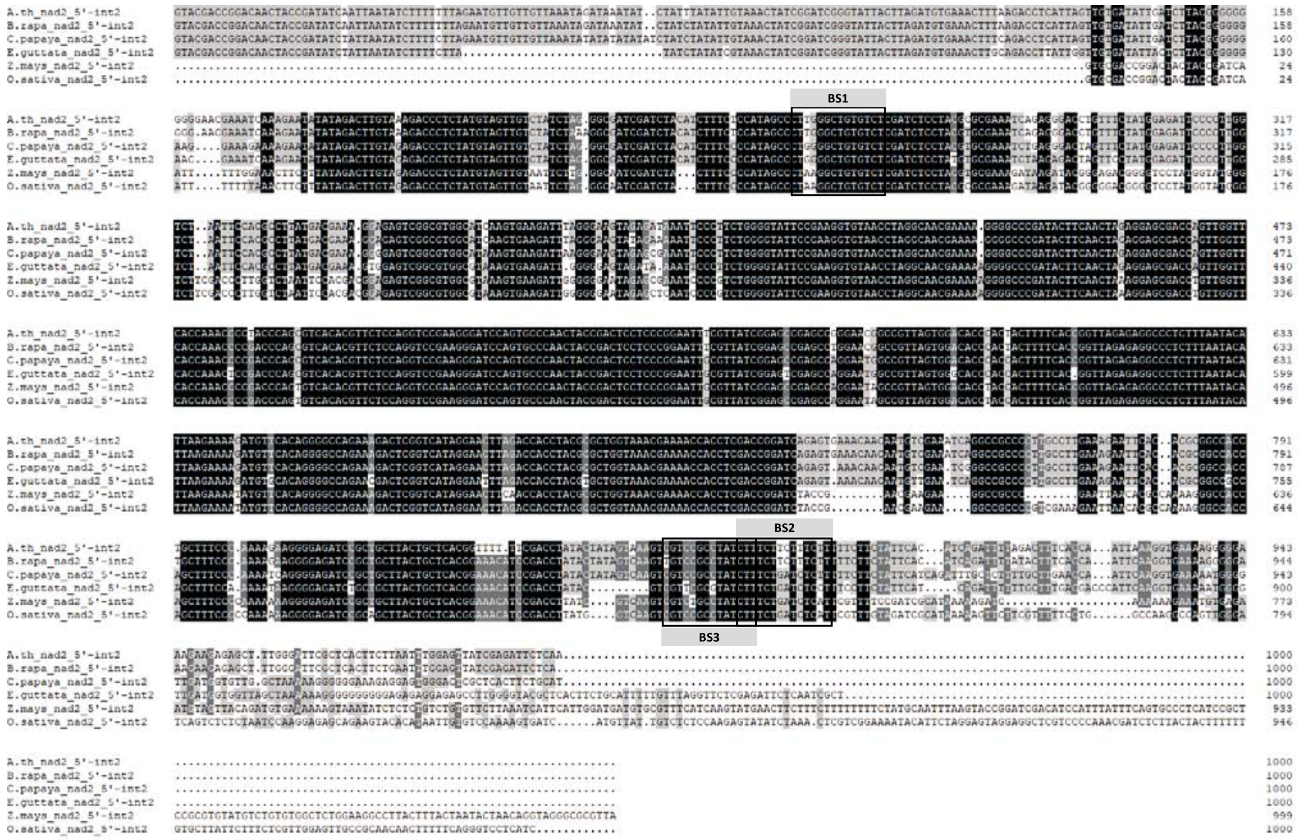

A.th_nad2_5'-int2
B.rapa_nad2_5'-int2
C.papaya_nad2_5'-int2
E.guttata_nad2_5'-int2
Z.mays_nad2_5'-int2
O.sativa_nad2_5'-int2
BS1
BS2
BS3

C

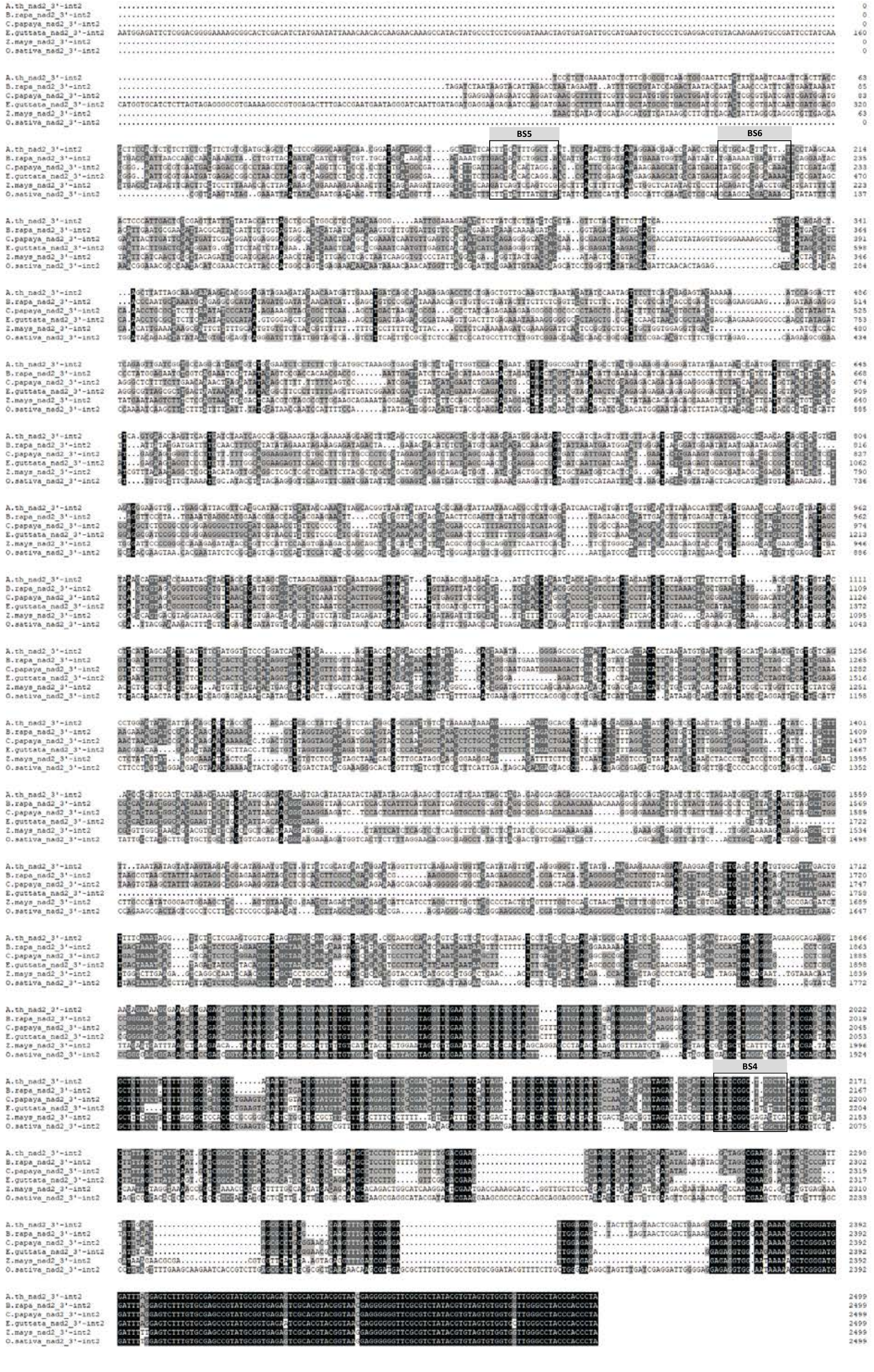

A.th_nad2_3'-int2
B.rapa_nad2_3'-int2
C.papaya_nad2_3'-int2
E.guttata_nad2_3'-int2
Z.mays_nad2_3'-int2
O.sativa_nad2_3'-int2
BS5
BS6
BS4

D

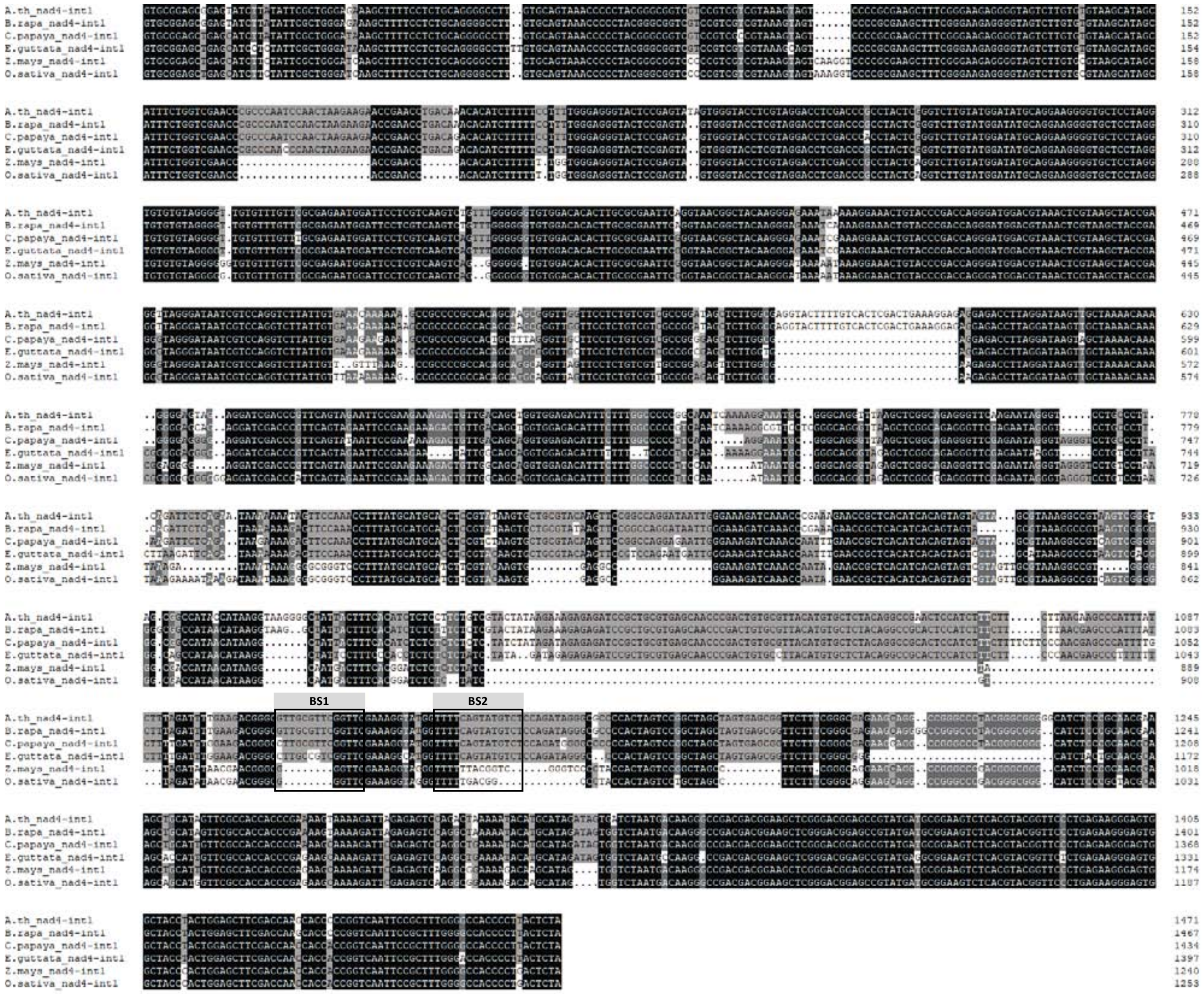
A.th_nad4-int1
B.rapa_nad4-int1
C.papaya_nad4-int1
E.guttata_nad4-int1
Z.mays_nad4-int1
O.sativa_nad4-int1
BS1
BS2

E

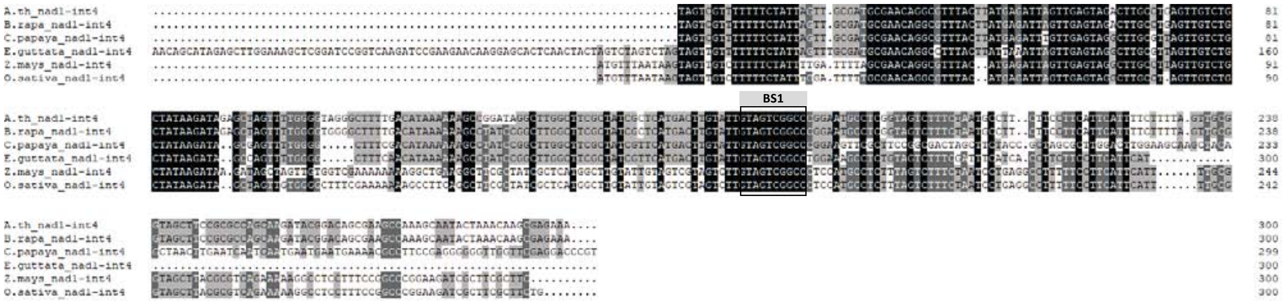

BS1
A.th_nad1-int4
B.rapa_nad1-int4
C.papaya_nad1-int4
E.guttata_nad1-int4
Z.mays_nad1-int4
O.sativa_nad1-int4

F

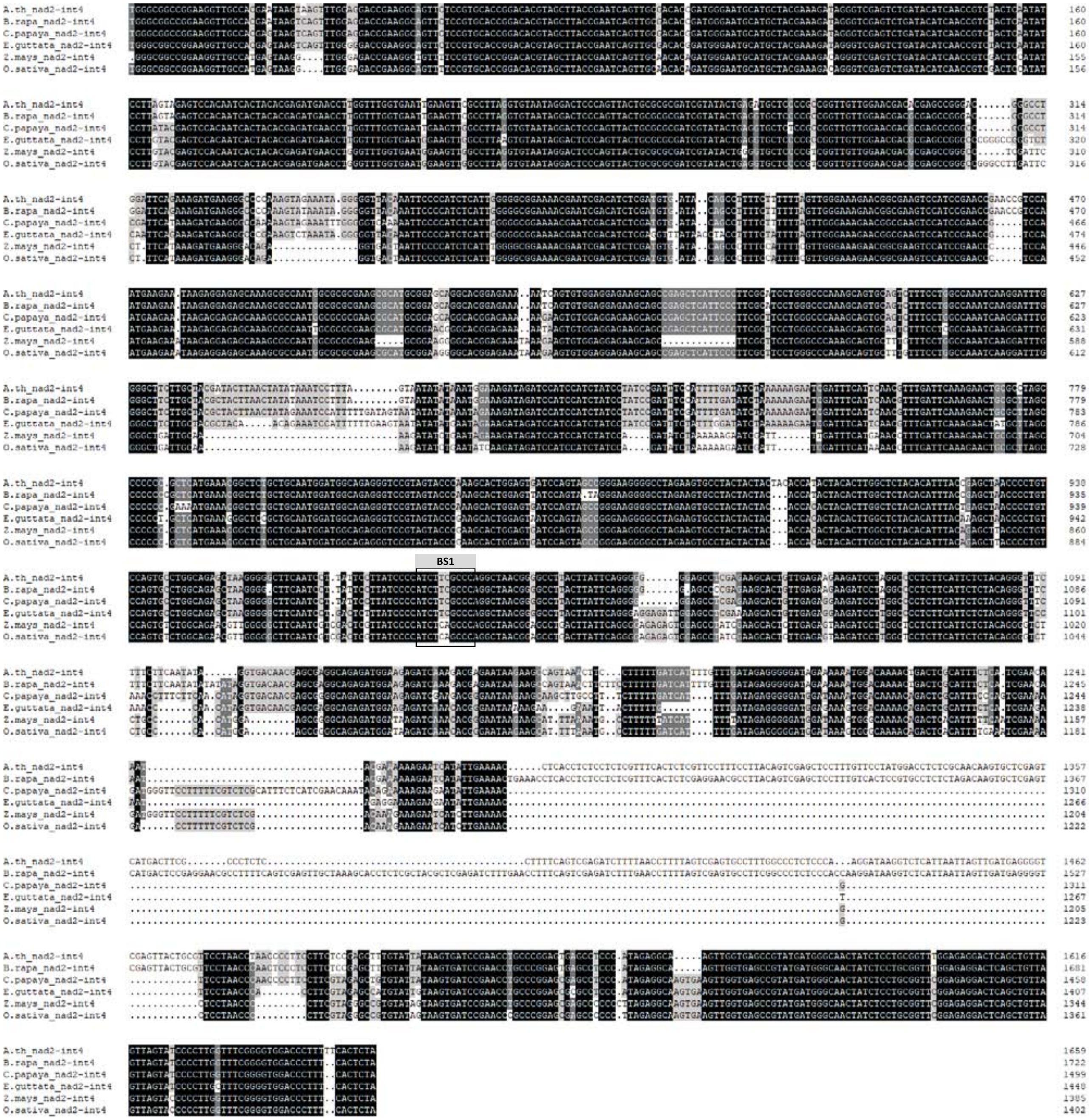

BS1
A.th_nad2-int4
B.rapa_nad2-int4
C.papaya_nad2-int4
E.guttata_nad2-int4
Z.mays_nad2-int4
O.sativa_nad2-int4

Supplementary Figure S7: Multiple sequence alignments analyzing the conservation of the different predicted RNA targets of each MISF protein. Multiple sequence alignments of the different MISF protein intron targets from a representative selection of dicot (*Arabidopsis thaliana*, *Brassica rapa* and *Carina papaya*) and monocot *(Oryza sativa*, *Zea mays*) species. The sequences corresponding to the MISF predicted binding sites are shown in black boxes. (A): Alignment of *nad2* intron 3 sequences. BS1 to BS3 represent the three putative RNA binding sites of MISF26 in *nad2* intron 3. (B) and (C): Sequence alignment of 5'- and 3'-portions of *nad2* intron 2 (trans-intron). The six potential RNA binding sites of MISF68 are shown as BS1 to BS6. (D): Sequence alignment of *nad4* intron 1. The two potential RNA binding sites of MISF68 are shown as BS1 to BS2. (E): Alignment of *nad1* intron 4 sequences. Since *nad1* intron 4 is fragmented in certain plant species, the sequences used for the alignment correspond only to the 300 nucleotides downstream of the *matR* open reading frame. The only putative RNA binding site of MISF74 is shown as BS1. (F): Alignment of *nad2* intron 4 sequences. BS1 represents the single putative RNA binding site found for MISF74 in *nad2* intron 4.
